# Advancements and Developments in Dietary Assessment Techniques

**DOI:** 10.3390/nu18030527

**Published:** 2026-02-05

**Authors:** Dazhou Zhu, Xiao Ren

**Affiliations:** Institute of Food and Nutrition Development, Ministry of Agriculture and Rural Affairs, Beijing 100081, China; rxiao1022@163.com

## 1. Introduction

With the continuous improvement in living standards, consumers are shifting their focus from merely achieving satiety and food safety to prioritizing nutrition and health, placing greater emphasis on the nutritional value of food and healthy dietary practices. However, despite this cognitive shift, nutrition-related health issues remain widespread and, in some cases, have even increased [1]. Poor dietary habits and nutritional imbalances are major preventable risk factors for non-communicable diseases (NCDs). In 2017, dietary risk factors were responsible for 11 million deaths and 255 million disability-adjusted life years (DALYs) (95% uncertainty interval: 234 to 274 million). Among these, high sodium intake (3 million deaths and 70 million DALYs), low intake of whole grains (3 million deaths and 82 million DALYs), and insufficient fruit consumption (2 million deaths and 65 million DALYs) were the leading dietary risk factors globally and in many individual countries [2]. The goal of personalized and precision nutrition is to maintain or improve health by leveraging human diversity through tailored dietary interventions, products, or services. To implement individualized interventions aimed at improving both population and planetary health, it is essential to measure and evaluate dietary intake [3].

For decades, the assessment of dietary intake and components has primarily relied on methods such as dietary records, 24 h dietary recalls, food frequency questionnaires, brief dietary assessment instruments, diet history, blended or combined instruments, dietary screening tools, and food frequency questionnaires [4]. In recent years, several technological advancements—such as image and video capture technologies, as well as other devices capable of measuring dietary intake and eating behaviors—have been widely adopted [5]. Scientifically analyzing and evaluating the nutritional value of foods and dietary patterns has become a key component of nutritional interventions and consumer guidance. The emergence of information technology, omics technologies, and novel model organisms has created new opportunities for the advancement and innovation of dietary assessment techniques. Dietary assessment in the context of precision nutrition requires not only consideration of an individual’s genomic background and gene–nutrient interactions but also a comprehensive phenotypic evaluation [6]. Approaches such as nutritional genetics, epigenetics, genomics, metabolomics, and metagenomics have laid a scientific foundation for understanding inter-individual variability in dietary responses and for advancing personalized and precision nutrition interventions [7]. At the same time, the development of big data and machine learning plays a crucial role in enabling the integration and application of precision nutrition strategies [1,3].

This Special Issue aims to explore emerging methods, models, technologies, and related applications in dietary and nutritional assessment. It features contributions that present approaches, models, and techniques for evaluating the nutritional quality of individual dietary components (such as raw ingredients and agricultural products), as well as methods for assessing the nutritional value of processed and prepared foods. It also includes studies on dietary surveys and methods, models, and technologies for evaluating dietary patterns and structures, along with research analyzing dietary habits across different regions and populations, and their associations with human nutrition and health. The following sections highlight two major themes that have emerged from the contributions to this issue, along with considerations for future research directions.

## 2. Assessment from the Perspective of Human Nutritional Needs

Pi-Hui Hsu et al. investigated the association between Total Dietary Quality Score (TDQS) and metabolic outcomes in Taiwanese adults with type 2 diabetes mellitus (T2DM). Their findings demonstrated that a higher TDQS was significantly associated with improved metabolic outcomes, supporting TDQS as a practical, multidimensional tool for clinical nutritional assessment and personalized dietary intervention (Contribution 1). Yuchao Feng et al. conducted an integrated metabolomic–transcriptomic analysis to examine the metabolic and genetic effects of three heat-stress-regulated mung bean polyphenols on Mode-K intestinal epithelial cells in mice under varying levels of heat stress. Their results confirmed the potential of mung bean polyphenols as a novel dietary intervention strategy to mitigate heat stress (Contribution 5). Another study evaluated the thirst-quenching effects of different beverages consumed prior to exercise, providing scientific evidence to guide the selection of the most appropriate beverage types (Contribution 4). In addition, Chia-Hui Lin et al. developed and validated a competency assessment questionnaire for medical assistants caring for older adults with dysphagia, aimed at evaluating their knowledge, attitudes, and behaviors regarding nutrition-focused dietary support (Contribution 3).

## 3. Assessment Enabled by Emerging Technologies

Portion size estimation is a critical component of dietary assessment and nutrition research, as it provides insights into the relationship between diet and health outcomes. Digital tools—such as food image recognition, artificial intelligence (AI) applications, and smartphone-based devices—have been utilized to develop a digital visual food atlas for Central Asian cuisine, offering high-quality images of commonly consumed foods and beverages (Contribution 2). Víctor de la O et al. integrated nutritional metabolomic data with machine learning approaches to evaluate the feasibility and effectiveness of using machine learning to analyze biomarkers for characterizing food and nutrient intake and predicting dietary patterns (Contribution 6). Yu Fan et al. conducted a systematic evaluation and comparison of GPS-assisted physical activity interventions combined with dietary interventions, assessing their practicality and effectiveness in improving adult obesity outcomes (Contribution 7).

## 4. Future Directions

Future research in dietary assessment is gradually advancing toward two key directions: (1) the development of multidimensional health evaluation systems based on individual nutritional needs, and (2) the integration of emerging information technologies for precise dietary data acquisition and intelligent analysis. As public awareness of the relationship between diet, nutrition, and chronic disease prevention continues to grow, traditional self-reported dietary assessment methods—such as 24 h recalls and food frequency questionnaires—face increasing limitations in terms of accuracy and efficiency. Consequently, future studies are expected to place greater emphasis on integrating multi-omics data—including metabolomics, nutrigenomics, epigenetics, and microbiomics—to explore interindividual variations in physiological responses to dietary interventions, thereby supporting the implementation of personalized and precision nutrition strategies. Meanwhile, rapid advancements in technologies such as artificial intelligence, image recognition, mobile devices, and wearable sensors are providing more objective and efficient tools for portion size estimation, food identification, and dietary behavior tracking. In addition, applying machine learning methods to construct predictive models linking dietary intake to health outcomes offers new pathways for large-scale nutritional surveillance and risk assessment. Future research should further promote the deep integration and intelligent processing of multi-source data, driving a shift in dietary assessment systems from static, fragmented records toward dynamic and continuous monitoring. This evolution will lay a solid foundation for achieving the full spectrum of goals—from individual health promotion and public health interventions to the development of sustainable food systems.
